# Use of dietary supplements among people living with HIV/AIDS is associated with vulnerability to medical misinformation on the internet

**DOI:** 10.1186/1742-6405-9-1

**Published:** 2012-01-10

**Authors:** Seth C Kalichman, Chauncey Cherry, Denise White, Miche'l Jones, Moira O Kalichman, Mervi A Detorio, Angela M Caliendo, Raymond F Schinazi

**Affiliations:** 1 Department of Psychology, University of Connecticut, 406 Babbidge Road, Storrs, CT 06269, USA; 2 Department of Pathology and Laboratory Medicine and Center for AIDS Research, Emory University School of Medicine, Medical Research Service #151-H, VA Medical Center, 1670 Clairmont Rd, 4900-001-1AA, USA; 3 Center for AIDS Research, Emory University School of Medicine and Veterans Affairs Medical Center, Medical Research Service #151-H, VA Medical Center, 1670 Clairmont Rd, 4900-001-1AA, USA

**Keywords:** HIV treatment, medical misinformation, treatment beliefs, dietary supplements

## Abstract

**Background:**

Use of dietary supplements is common among people living with HIV/AIDS. Because dietary supplements are used in the context of other health behaviors, they may have direct and indirect health benefits. However, supplements may also be associated with vulnerability to medical misinformation and unfounded health claims. We examined use of dietary supplements among people living with HIV/AIDS (PLWH) and the association between use of dietary supplements and believing medical misinformation.

**Methods:**

A convenience sample of 268 men and 76 women living with HIV was recruited from AIDS services and clinics in Atlanta, GA. Participants completed measures of demographic and health characteristics, dietary supplement use, beliefs about dietary supplements, internet use, and an internet evaluation task designed to assess vulnerability to medical misinformation.

**Results:**

One out of four PLWH currently used at least one dietary supplement product excluding vitamins. Dietary supplement use was associated with higher education and greater use of the internet for health-related information. Dietary supplement users also endorsed greater believability and trust in unfounded claims for HIV cures.

**Conclusions:**

Dietary supplement use is common among PLWH and is associated with a broad array of health information seeking behaviors. Interventions are needed to reduce the vulnerability of PLWH, particularly dietary supplement users, to medical misinformation propagated on the internet.

## Introduction

Dietary supplements play an important role in the lives of many people living with chronic and often life-threatening medical conditions. Dietary supplements generally consist of diverse products that are typically not part of current mainstream, conventional health care [1]. Concerns about dietary supplements generally stem from the potential for adverse interactions with conventional medicines and patients replacing evidence-based health care with untested remedies[2]. Studies show that dietary supplements are often used by people living with HIV infection. The most common dietary supplements in people living with HIV/AIDS (PLWH) are used to 'boost immune functioning' such as mega-dose vitamins, and anti-oxidants and body cleansing products such as teas and herbs to remove 'toxins'[3]. As many as half of PLWH may use complementary and alternative medicines, which can include dietary supplements [4].

Dietary supplement users tend to engage in a range of health promoting behaviors [3,5]. Studies of cancer patients show that actively seeking health information from multiple sources is a associated with dietary supplement use [6]. It is common for PLWH who use dietary supplements to conceal these practices from their health care providers, potentially increasing the risks for interference with treatment plans[5]. PLWH who use dietary supplements with the potential for adverse drug interactions are also inclined to seek health information from a variety of sources [4], including non-traditional and fringe groups, suggesting a vulnerability to misinformation and fraudulent claims.

Access to credible health information on the internet can be an important part of patient-centered health care. Unfortunately, the internet is also a major source of medical misinformation and unconventional health promotion. Incomplete and inaccurate health information is common on the internet and for many patients unreliable information sources are indistinguishable from credible ones [7-10]. Unproven cancer treatments promoted online, for example, cause significant concern and have led to measures that counter their use [11]. Similarly, South African courts have banned the promotion of vitamins to cure AIDS [12-14], after years of devastating government support for fake AIDS treatments [15-17]. Anecdote-based remedies are appealing to patients with serious health conditions because they speak in certain terms about their successes, emphasize the uncertainties and side-effects of conventional medicine, prey upon anxieties, and offer unrealistic hope [6]. Among PLWH, unsubstantiated claims by marketers of 'immune boosting products' and fake cures are often seen as credible and evoke interest in their use [9]. However, we are not aware of research that has investigated vulnerability to medical misinformation and fake claims among dietary supplement users living with HIV/AIDS.

The purpose of the present study was to examine dietary supplement use among PLWH and its association to vulnerability to medical misinformation. First, we describe past and current use of dietary supplements in a community sample of PLWH. We then describe the demographics, health characteristics, and information seeking behaviors of current dietary supplement users compared to persons not using dietary supplements. Finally, we tested the potential vulnerability to medical misinformation among active dietary supplement users. We focused on responses to claims that HIV/AIDS can be effectively treated and cured by dietary supplements. We hypothesized that PLWH who are using dietary supplements would be more interested in and believe the claims of medical misinformation propagated on the Internet.

## Methods

### Participants

People living with HIV/AIDS were recruited through targeted community sampling to participate in a cross-sectional study. We used both targeted venue recruitment and snowball sampling techniques to identify individuals in and out of care. Recruitment relied on responses to brochures placed in waiting rooms of HIV service providers and infectious disease clinics throughout Atlanta, GA. We also implemented an explicit systematic approach to word-of-mouth chain recruitment. Specifically, participants were given brochures that describe the study opportunity with a phone number to the research offices. Participants were encouraged to use the brochures to refer their HIV-infected friends to the study. These procedures were designed to extend recruitment beyond service settings in order to achieve a broad community sample of people living with HIV/AIDS. The entry criteria were age 18 years or older, scored at least 80% correct on a test of functional health reading literacy (TOFHLA) [18] and showed proof of their positive HIV status.

### Measures

Measures were collected at the research site using an instructor guided self-administration procedure in groups of 4 to 8 persons. Participants were shown page by page how to complete the measures by using a projected facsimile, assuring that instructions for each instrument were carefully described and that participants were given privacy when responding. Data were collected between January 2008 and June 2009. Written informed consent was obtained from the participants of this study and the study was approved the university Institutional Review Board.

#### Demographic and health characteristics

Participants were asked their age, years of education, income, ethnicity, HIV treatment history, and employment status. HIV related symptoms were assessed using an adapted version of a previously developed measure [19], which included 14 common symptoms of HIV infection, including developing a new skin rash, recurring fever, chronic diarrhea, and persistent shortness of breath. We asked participants to provide blood specimens to test for HIV (RNA) viral load. Blood samples were provided at the project offices using standard phlebotomy and couriered to the lab for processing. Whole blood specimens in EDTA tube (Becton Dickinson) were centrifuged at 500 *g *for 10 min within 4 hrs of collection. The plasma was recovered and aliquoted into 1 ml samples and stored at -70°C. Plasma viral load was determined by Roche Amplicor HIV-1 Monitor.

Participants consented to monthly unannounced telephone-based pill counts, constituting a prospective measure of adherence. Unannounced pill counts are reliable and valid in assessing HIV treatment adherence when conducted in participants' homes [20] and on the telephone [21,22]. Participants were provided with a cell phone that restricted service for project contacts and emergency use (e.g., 911). Following office-based training in the pill counting procedure, participants were called at unscheduled times by a phone assessor. Pill counts occurred over 21 to 35 day intervals and were conducted for each of the medications that participants were taking. Pharmacy information from pill bottles was also collected to verify the number of pills dispensed between calls. Adherence was calculated as the ratio of pills counted relative to pills prescribed, taking into account the number of pills dispensed. Two consecutive pill counts were necessary for computing adherence monitored for eight consecutive months. This study used the mean value of all available adherence data points as a stable indicator of HIV treatment adherence.

#### Dietary Supplements

We created an assessment of commonly used dietary forms of complementary medicine. We derived items for this measure through 3 sources. First, we selected dietary supplements described by the National Center for Complementary and Alternative Medicine. [Antioxidants, Chamomile & other teas, Flax seed, Macrobiotics, Probiotics, Selenium, St. Johns Wort] [1] Second, we identified additional items from previous research on the frequency of dietary supplement use among PLWH [Herbs] [4,23] Finally, we held informal discussions with ten people living with HIV who participated in past research regarding their experience with dietary supplements. [Amino acid therapy, Immunotherapy, Micronutrient therapy, Orange juice pills, Chinese medicine, African medicine] The final result was 14 dietary supplements. Participants were presented with the list of dietary supplement practices and instructed to mark each for whether they (a) had ever used it since testing HIV positive and (b) whether they were currently using it. We also asked participants who reported current use of dietary supplements to estimate the amount of money they spend per month on these products.

#### Dietary Supplement Beliefs

We assessed participants beliefs regarding the benefits of dietary products for curing HIV infection and AIDS. Participants completed 4 items regarding vitamins, traditional and herbal remedies, and immune boosters. The exact items are shown in the results section. Items were responded to as 'Agree' and 'Disagree'.

#### Internet use for health-related information

Participants indicated whether they had used the Internet to find health-related information, purchase health products, and shared health information they had accessed online. Participants reported the number of times they had used the Internet for these purposes in the previous month. Open responses were collected by participants writing values in blank spaces for the number of times they performed each action.

#### Perceptions of e-health information

We assessed perceptions of unsubstantiated claims available on the internet regarding the use of dietary practices to treat and cure AIDS. Participants completed an internet rating task adapted from previous research [24,25]. For this task, webpages were obtained directly from the Internet, including color, image resolution, and text size. We selected two webpages representing false claims for using micronutrients to treat and cure AIDS: (a) Rath International: "*Micronutrients Help Control AIDS" *http://www4.dr-rath-foundation.org/pdf-files/ri_2006_02_en.pdf; summarizes the findings from uncontrolled studies that have been deemed unauthorized and illegal[12,13]. The specific webpage reports 'clinical proof' that micronutrients improve the health of PLWH and that antiretroviral medications are toxic and without benefit; (b) Jonathan Campbell; *"A Cure for AIDS?" *http://www.cqs.com/aidscure.htm endorses the use of "immune system enhancing nutrients such as vitamin C (in absolutely massive doses) and zinc". This webpage states that the pharmaceutical industry promotes "drugs such as AZT that focus on destroying HIV (meanwhile killing the patient)".

One reputable website was included as a control: Tufts School of Medicine "*Choose Snacks that work for you" *http://www.tufts.edu/med/nutrition-infection/hiv/health_snacks.html; explains the nutritional value of healthy snacks and the benefits from healthy food choices.

As a check for whether participants had read the internet passages, we asked three factual true/false questions extracted from the text of each passage. Participants also indicated their perceptions of and interest in the three sources of information by rating four dimensions: "How much do you believe this information?", "How factual is the information?", "How much do you trust the information?" and "How important is this information for you?" using 10-point scales ranging from 1 = 'Not at all' to 10 = 'Very much'.

### Data analyses

We performed descriptive analyses to examine the frequency of past and current use of 14 dietary supplements separately for men and women. We then compared current dietary supplement users (N = 93) to PLWH who were not currently using dietary supplement (N = 251) on demographics, health characteristics, internet use, perceptions of website information, and dietary supplement beliefs. Comparisons on continuous measures used independent groups t-tests and categorical variables were analyzed using contingency table chi-square tests. We performed a final multivariable logistic regression analysis to test the independent effects of factors related to current dietary supplement use. This multivariable model simultaneously tested non-overlapping factors from the previous analyses found significantly associated with current dietary supplement use. To avoid statistical redundancy we created a total index of interest and believability of the Rath and Campbell website claims regarding treating and curing AIDS. Specifically, we calculated the mean score of the four interest and believability ratings. In addition, we created a composite of the four dietary supplement beliefs by summing the endorsements, ranging from 0 endorsed to 4. From the logistic regressions we report odds ratios with 95% confidence intervals. All analyses used case-wise deletion for missing values and defined statistical significance as p < .05.

## Results

A convenience sample of 268 men and 76 women was recruited from AIDS services, health care providers, social service agencies, and infectious disease clinics in Atlanta, GA. Results showed 26% (N = 93) currently used at least one dietary supplement. (see Table 1) The most commonly used dietary products were antioxidants and teas. Seventy percent (N = 65) of dietary supplement users reported currently using two or more products. There were no statistically significant differences in dietary supplement use between genders; men used an average of 0.82 (SD = 1.8) dietary supplement products compared to women who used 0.60 (SD = 1.45) products, t(df = 342) = 0.3. Among persons who reported current use of dietary supplements, men spent an average $49.85 and women spent $34.29 per month on these products, a non-significant difference, t(df = 343) = 0.5.

**Table 1:** Current and past dietary supplement product use among 268 men and 76 women living HIV/AIDS.

	Current Supplement Use				Past Supplement Use			
	Men		Women		Men		Women	
Products	N	%	N	%	N	%	N	%
Antioxidants	48	18	12	16	37	14	8	11
Amino acid therapy	8	3	0	0	15	6	2	3
Chamomile & other teas	48	18	10	13	27	11	6	8
Flax seed	18	8	5	6	20	8	1	1
Herbs	40	15	8	11	39	15	6	8
Immunotherapy	7	2	2	2	16	6	3	4
Macrobiotics	4	2	0	0	5	2	0	0
Micronutrient therapy	9	3	2	2	8	3	0	0
Orange juice pills	9	3	1	1	12	5	1	1
Probiotics	3	1	2	2	7	3	2	2
Selenium	12	5	4	5	18	7	1	1
St. Johns Wort	3	1	0	0	13	5	1	1
Chinese medicine	5	2	0	0	9	3	2	2
African medicine	4	2	0	0	3	1	2	2
**Number of products in current use**								
**None**	190	71	61	80				
**1**	25	9	3	4				
**2-3**	29	11	6	8				
**4+**	24	9	6	8				

### Demographic and health characteristics

Results showed that dietary supplement users had significantly more years of education. (see Table 2) There were no other significant associations between demographic characteristics and supplement use. Although dietary supplement users tended to have higher medication adherence the difference was not statistically significant. There were no other significant differences between dietary supplement non-users and users on health characteristics.

**Table 2:** Demographic and health characteristics of people living with HIV/AIDS who do not use and use dietary supplements.

	Do Not Use Supplements (n = 251)		Uses Supplements (n = 93)			
Characteristic	M	SD	M	SD	t	p
Age (years)	44.5	7.4	44.0	8.7	0.5	n.s.
Education (years)	12.3	1.9	13.4	1.9	4.7	.01
Monthly income ($)	1104	3301	1029	1322	0.2	n.s.
Years since HIV+	13.0	7.2	13.8	7.0	0.9	n.s.
CD4-cell count (cells/mm^3^)	445	314	516	320	1.6	n.s.
HIV symptom score	3.8	3.6	4.4	4.0	1.1	n.s.
Number of AIDS diagnoses	1.9	4.8	1.7	3.5	0.4	n.s.
ART adherence (%)	79.5	22.2	85.0	16.3	1.6	n.s.
	N	%	N	%	*X*^2^	p
Men	190	76	78	83		
Women	61	24	15	17	2.6	n.s.
African American	233	93	84	90		
White	14	6	4	5		
Other race	3	1	5	5	5.3	n.s.
Viral load < 50 copies	102	47	48	59	2.9	n.s.
Adherence > 85%	69	52	30	56	0.2	n.s.

### Internet use for health-related information

Dietary supplement users demonstrated significantly greater use of the internet with respect to searching for medical, treatment, and health information online. (see Table 3) Dietary supplement users were also significantly more likely to purchase health-related products in general and share information obtained online with their friends. Thus, dietary supplement users indicated significantly greater internet use for locating and sharing health-related information.

**Table 3:** Internet use in the previous month among people living with HIV/AIDS who do not use and do use dietary supplements.

	Do Not Use Supplements (n = 251)		Uses Supplements (n = 93)			
Internet use in previous month	N	%	N	%	*X*^2^	p
Any use of the Internet	116	46	73	79	29.6	.01
Searched HIV treatments	38	15	38	41	26.6	.01
Purchased health products	7	3	9	10	7.4	.01
Search medication information	35	14	28	30	12.2	.01
Search general health information	51	20	49	53	35.2	.01
Shared information from internet with friend	62	24	46	50	19.9	.01
Friend shared information from internet	52	21	42	45	21.0	.01

### Vulnerability to false claims about dietary supplements and supplement beliefs

As an internal validity check, we first analyzed responses to the factual recall questions asked for each internet passage. Overall accuracy for identifying information contained in the website passages was high, with over 80% correct responses across the three passages. However, dietary supplement users were significantly more likely to correctly report the website content, 87.7% (SD = 13.4), compared to non-dietary supplement users, 78.0% (SD = 17.8), t(df = 343) = 4.9, p < .01.

Results for the website information ratings are shown in Table 4. Comparisons indicated that in every case, for all three internet passages, dietary supplement users were significantly more inclined to believe the information, endorse its factual basis, trust it, and endorse its personal importance. This same pattern of higher interest and believability of internet health information was observed for the two false claims about treating and curing AIDS with vitamins and micronutrients as well as the control website concerning choosing healthy snacks.

**Table 4:** Ratings of perceived HIV treatment and cure information found on the Internet among people living with HIV/AIDS who do not use and do use dietary supplements.

	Do Not Use Supplements (n = 251)		Uses Supplements (n = 93)			
Internet source and perception rating	M	SD	M	SD	t	p
**Matthias Rath - "Micronutrients Help Control AIDS"**						
Believes the information	6.4	2.6	4.2	2.6	2.3	.05
Information is factual	6.4	2.7	7.2	2.3	.05	
Trust the information	6.3	2.7	7.0	2.8	1.9	n.s.
Information is important to me	6.9	2.9	7.8	2.8	2.3	.05
**Jonathan Campbell - "A Cure for AIDS?"**						
Believes the information	6.2	2.8	7.5	2.6	3.6	.01
Information is factual	6.1	2.8	6.8	2.8	2.2	.05
Trust the information	5.8	2.8	6.9	2.8	3.4	.01
Information is important to me	7.6	3.0	8.5	2.6	2.4	.01
**Mean ratings Rath and Campbell scenarios**	6.5	2.3	7.3	2.2	3.3	.01
**Tufts Medical School - "Choose Snacks That Work for You!"**						
Believes the information	7.9	2.4	8.9	1.9	3.6	.01
Information is factual	7.5	2.6	8.6	2.1	3.6	.01
Trust the information	7.6	2.5	8.7	2.0	3.6	.01
Information is important to me	8.2	2.4	8.8	2.4	2.0	.05

A similar pattern of results emerged for endorsing beliefs that vitamins and natural remedies can treat and cure AIDS; dietary supplement users were significantly more likely to believe vitamins, healthy foods, and traditional medicines can cure AIDS. (see Table 5) Overall, dietary supplement groups significantly differed in the total number of dietary supplement beliefs endorsed, t(df = 343) = 4.29, p < .01.

**Table 5:** Dietary supplement beliefs among people living with HIV/AIDS who do not use and do use dietary supplements.

	Do Not Use Supplements (n = 251)		Uses Supplements (n = 93)			
Beliefs	N	%	N	%	*X*^2^	p
Vitamins and healthy foods can cure AIDS.	24	9	23	25	13.6	.01
Traditional medicines can cure AIDS.	12	5	13	14	8.7	.01
Herbal and natural remedies can cure AIDS in some people.	69	28	40	43	7.5	.01
HIV is treatable using non-toxic natural immune boosters.	100	40	47	51	3.4	n.s.
Overall mean (SD) items endorsed^a^	0.8	(0.9)	1.1	(1.1)	4.84	.01

Note: Mean perception ratings on 10-point scales, 1 = 'Not at all' to 10 = 'Very much', ^a ^t-value for comparison between group means

### Multivariable model

Table 6 presents the results of the multiple logistic regression predicting current dietary supplement use from non-redundant participant characteristics. Results showed that being younger and better educated, having an undetectable viral load at the time of the study, and using the Internet for health information and rating false treatment information as more interesting and believable were independently associated with the use of dietary supplements.

**Table 6:** Multiple logistic regression predicting current dietary supplement use.

Predictor	Odds Ratio	p	95%CI
Age	.94	.01	0.91-0.98
Education	1.47	.01	1.25-1.73
Years since testing HIV positive	1.04	n.s.	1.00-1.09
HIV symptoms	1.05	n.s.	0.97-1.13
Undetectable viral load	2.35	.01	1.26-4.36
Searched for health information online	1.07	.01	1.01-1.13
Interest and believability of internet medical misinformation	1.23	.01	1.08-1.41
Beliefs that supplements can treat and cure AIDS	1.41	.05	1.06-1.89

## Discussion

Results of the current study demonstrated that one in four participants currently used at least one dietary supplement product. The overall use of dietary supplements in this sample was similar to past research with other medical populations. For example, a study of chronic lymphocytic leukemia patients found that 44% had used dietary supplements [26]. Previous research with PLWH also demonstrated similar use, such as a study of HIV positive men that found 69% used complementary medicine products and practices, the most common of which were dietary supplements [4].

The current study found that age and years of education were the only demographic characteristics associated with dietary supplement use; individuals who used dietary supplements were younger and significantly better educated. With respect to health markers, dietary supplement users were more likely to have an undetectabale viral load. It is important to note that while some dietary supplements can adversely interact with prescriprition medications, some supplements may also have positive impacts on HIV disease processes. For exampe, recent research suggests that selenium, which was used by 5% of our sample, may have benefits in treating HIV infection [27].

In the current study there was no association between supplement use and medication adherence, suggesting that participants may consider dietary supplements as co-treatments that do not interfere with their medications. Consistent with past research, we also found that dietary supplement use was associated with an array of health-related behaviors. In this case, dietary supplement users were significantly more likely to use the Internet for health-related information seeking. In addition to actively seeking health information on the internet, dietary supplement users also demonstrated greater trust in internet information and more positive beliefs in the curative value of dietary supplements. In addition, dietary supplement users were more inclined to believe and trust both unfounded claims as well as credible health information. These results confirm our main study hypothesis to show that dietary supplement use is associated with accepting medical misinformation. Our cross-sectional study, however, does not allow us to infer directions of the relationships. Specifically, people who use dietary supplements may be more vulnerable to misinformation or misinformed individuals may be more prone to using dietary supplements. These results also replicate previous research that has found high-rates of trust and believability in false claims on the internet among PLWH [23,24]. Taken together, our findings suggest that dietary supplement use may be a marker for health seeking behaviors and an openness to using diverse treatment approaches regardless of their evidence-base, suggesting a vulnerability to quackery and fraud.

The findings from this study should be interpreted in light of its methodological limitations. With the exception of viral load assessed by blood labs and medication adherence monitored using an objective assessment, our study methods relied on self-reported health status and behaviors. As noted above, our cross-sectional study design precludes any causal or directional conclusions. The content of our measures may also have influenced our findings. Specifically, our measure of dietary supplement beliefs did not necessarily include items most central to decisions about using or not using dietary supplements. Our measures also may have excluded important covariates that could help explain the results, such as illicit substance use and medication side effects. It is also unknown whether responding to the study measures inadvertently stimulated interest or reinforced interest in dietary supplements. Finally, our results are based on a convenience sample that is predominantly middle-aged, African American, and from one southern US city. In addition, our recruitment procedures may have biased the sample toward recruiting networks of people who use dietary supplements. Although our results converge with other studies, caution is warranted before generalizing these findings to other populations of PLWH.

Dietary supplements play important roles in the health practices of many PLWH. Dietary supplement use often occurs in the context of antiretroviral therapies. Patients who gravitate toward alternative treatments should be counseled on the potential for misinformation and fraud on the Internet. The value of legitimate dietary supplements can be undermined by fraudulent and confusing medical misinformation on the Internet. Patient education and counseling about dietary supplement practices should occur while respecting well-informed individual choices in health care. Interventions that teach patients health consumer and critical thinking skills have demonstrated positive outcomes, including reduced vulnerability to internet-based misinformation [10]. Wide-scale use of the internet and the proliferation of e-health products challenges providers to discuss unfounded claims as well as the relative value of dietary supplements with their patients.

## Competing interests

The authors declare that they have no competing interests.

## Authors' contributions

SCK conceptualized the study, contributed to the data analyses, and prepared the manuscript. CC managed the study and implemented the study protocol. DW conducted interviews and performed quality assurance for interview data. MJ conducted interviews and performed quality assurance for interview data. MOK implemented the study protocol, managed the pill count data, and contributed to the study design.

MD performed laboratory analyses HIV data. AMC oversaw quality assurance and laboratory management for HIV data. RFS oversaw quality assurance and laboratory management of HIV data and contributed to the overall conceptualization of the study. All authors have read and approved the final manuscript.
